# Docking predictions based *Plasmodium falciparum* phosphoethanolamine methyl transferase inhibitor identification and in-vitro antimalarial activity analysis

**DOI:** 10.1186/s13065-019-0551-5

**Published:** 2019-03-28

**Authors:** Jagbir Singh, Rani Mansuri, Sonam Vijay, Ganesh Chandra Sahoo, Arun Sharma, Mahesh Kumar

**Affiliations:** 1 Division of Protein Biochemistry and Structural Biology, National Institute of Malaria Research (ICMR), Sector 8, Dwarka, New Delhi 110 077 India; 2 School of Pharmaceutical Sciences, Apeejay Stya University, Gurugram, India; 3 Division of ECD, Indian Council of India, New Delhi, India; 4 Department of Biomedical Sciences, Rajendra Memorial Research Institute, Patna, India; 5 Department of Pharmaceutical Sciences, Maharshi Dayanand University, Rohtak, India

**Keywords:** Phosphatidylcholine, *Pf*pmt, ADMET, Docking, Invitro, HEK-293

## Abstract

The increased multidrug resistance among antimalarial drugs produces the urgency of potent anti malarial to combat resistant malaria and the malaria burden worldwide. The protein which may prevent the growth or transmission of malaria parasite may be the great target for rational drug designing. *Plasmodium falciparum* phosphoethanolamine methyltransferase (*Pf*pmt) absent in human catalyzes triple methylation of ethanolamine into phosphocholine for phosphatidylcholine biosynthesis from serine decarboxylation phosphoethanolamine methyltransferase pathway for the membrane development at asexual as well as sexual stages of parasite. The *Plasmodium* requires production of membrane rapidly for growth and multiplication. Hence, the phosphoethanolamine methyltransferase of *Plasmodium falciparum* was selected as drug target for rational drug designing. Using Discovery studio 3.5 software the library of zinc compounds was screened against target and analyzed. The compounds with better druglike properties and docking affinity and binding interaction for target protein were procured for in vitro analysis against *Plasmodium falciparum* culture (IC_50_). Compounds ZINC02103914 and ZINC12882412 were found to have good druglike properties and affinity for *Pf*pmt also inhibited *P. falciparum* growth at very low µM IC_50_ concentration 3.0 µM and 2.1 µM respectively also found nontoxic in vitro against HEK-293 cells. Simulation study of best inhibitor revealed the specificity for the target protein. Hence, the compounds possessed the immense probability of being inhibitors of *Pf*pmt and may be optimized as antimalarial agent for combinational therapy to overcome the multidrug resistance and may also be used as template for optimization and rational drug designing.

## Background

Malaria is a mosquito borne disease in humans caused by unicellular microorganism called parasitic *Plasmodium* protozoa. According to the WHO, million new malaria cases were found and people were killed by malaria globally [1].

Malaria treatment recommended by WHO, must be followed by government as well private health organizations. Once the suspected cases are diagnosed with malaria, the treatment should be started according to the WHO guidelines for *Plasmodium falciparum*, *vivax* and others. Uncomplicated *P. falciparum* malaria treatment should be started with artemisinin combination therapy (Table 1). Since *Plasmodium falciparum* malaria can more be complicated than *P. vivax* infection, so extra attention is needed while observing the patient and should be treated as per drug policy. Patients with severe malaria should be given artesunate through intravenous or intramuscular route for at least 24 h or until patient become in condition to take oral medication. After 24 h and patient can tolerate medication orally, artemisinin combination therapy (ACT) treatment should be given for 3 days, (primaquine dose can be added in case of low transmission). Artemether should be preferred over quinine if artesunate (intravenous and intramuscular) is not available. In case of pre referral treatment when complete treatment of severe malaria is not available, in that case patient (children/adult) should be given Intramuscular artesunate and refer for complete treatment. But when both injection of artesunate and complete treatment are not available, in that case rectal dose of artesunate should be given to children < 6 years (avoid in older children and adults) and refer for complete treatment. *Plasmodium ovale* and *malariae* are rarely found in India, however *P. ovale* and *P. malariae* infection should be treated as *P. vivax* and *P. falciparum* [2, 3] respectively as given in Table 1. Since, the resistance against chloroquine and artemisinin has increased so *Plasmodium falciparum* infection cases should be treated with ACT because it is combination of drugs with different mechanism of action to block parasite development.

**Table 1:** Current treatment of *Plasmodium* malaria infection

*P. falciparum* Uncomplicated	*First*-*line treatment* Artesunate + sulfadoxine pyrimethamine 50 mg or 100 mg of artesunate and tablets containing 500 mg of sulfadoxine + 25 mg of pyrimethamine Artesunate + mefloquine/amodiaquine Recommended if the use of sulfadoxine–pyrimethamine (SP) is contraindicated. Artesunate 15–20 mg/kg and mefloquine 8 (5–11) mg/kg/dose daily for 3 days Primaquine (0.25 mg base/kg body weight, maximum dose 15 mg) is recommended for uncomplicated *P. falciparum* malaria as gametocytocidal *Second*-*line treatment* Artemether–lumefantrine Available as tablets coartem (20 mg artemether and 120 mg lumefantrine) twice daily for 3 days Primaquine (0.25 mg base/kg body weight, maximum dose 15 mg) is recommended for uncomplicated falciparum malaria as gametocytocidal *Third line treatment* In case of contraindication with first line and second line the third line treatment is recommended as Atovaquone 250 mg/proguanil 100 mg (malarone) tablets daily for 3 days Quinine + doxycycline for 7 days Quinine and clindamycin is the preferred for pregnant women during first trimester
*P. falciparum* (Special risk groups)	Pregnancy (first trimester): quinine + clindamycin for 7 days Infants (< 5 kg body weight): ACT (mg/kg) as given to the children of 5 kg body weight Malaria co-infected with HIV: avoid artesunate + sulfadoxine pyrimethamine if patient is on clotrimazole Avoid artesunate + amodiaquine if patient is on efavirenz or zidovudine
*P. falciparum* Severe malaria	Artesunate through intravenous (IV) or intramuscular (IM) route for atleast 24 h or until patient become in condition to take oral medication. After 24 h ACT treatment for 3 days (primaquine can be added) In absence of IV/IM artesunate, artemether should be preferred
*P. vivax*	*P. vivax*: chloroquine 25 mg for 3 days, in combination with primaquine 0.25 mg/kg and 15 mg for adults for 14 days in case of chloroquine-sensitive *P. vivax* malaria. Other ACTs should be combined with primaquine for chloroquine-resistant
*P. ovale*	*P. ovale*: treatment recommended for *P. ovale* malaria infection is same as for *P. vivax* malaria
*P. malariae* and *knowlesi*	*P. malariae* and *P. knowlesi*: infection is recommended for chloroquine like *P. vivax* malaria. No radical cure is required since, no hypnozoites are formed in infection

As the world is facing problem of antimalarial drug resistance is a greatest challenge today to stop the spread of malaria to new areas and re-emergence of malaria [4]. Hence, there is need for new drug target to develop potential inhibitors against the disease. So, a novel drug target *Plasmodium falciparum phosphoethanolamine methyltransferase* gene that is devoid in humans was selected to screen potent antimalarial drugs. Phosphatidylcholine is most frequently needed phospholipid for the survival of the parasites. *Plasmodium* synthesizes phosphatidylcholine abundantly from serine decarboxylation phosphoethanolamine methyltransferase pathway (SDPM) for production of new membranes at very fast rate for rapid multiplication during not only intraerythrocytic cycle (with maximum expression at trophozoite stage) but also during gametocyte development [5].

**Fig. 1:**
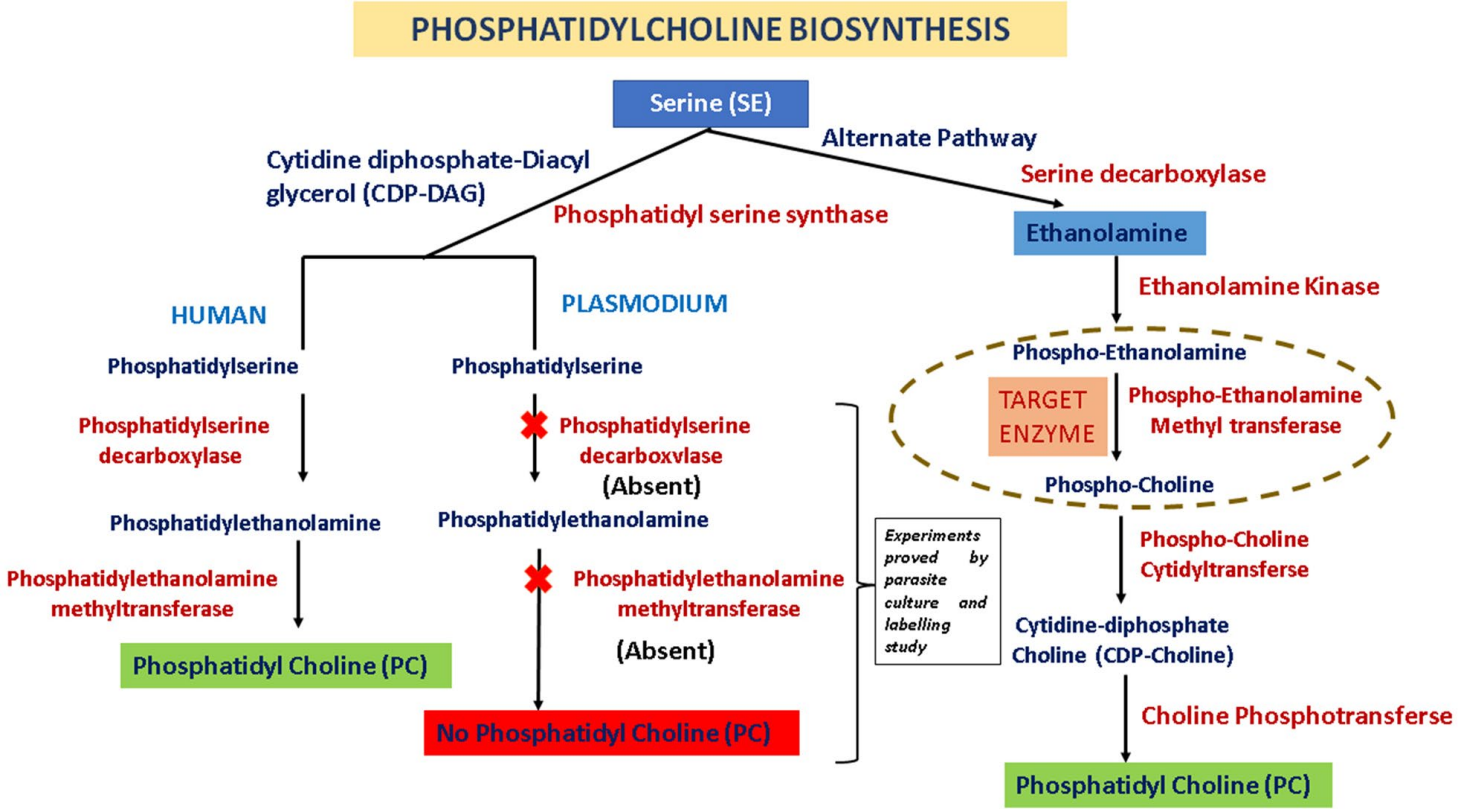
SDPM pathway a different route for PC in *P. falciparum* in comparison with human predominant PC synthesis. Plant-like reactions are indicated as in dotted circle. *DAG* diacylglycerol, *CDP* cytidine diphosphate, *PC* phosphatidylcholine

Loss of *Pf*pmt could severely distort the multiplication and gametocytes formation but reintroduction of *Pf*pmt effects were restored to wild type. *Pf*pmt has one catalytic domain for three methylation steps this implies that same catalytic domain is used for all three methylations (Fig. 1). Therefore, the inhibition of catalytic domain of *Pf*pmt may lead to complete inhibition of SDPM pathway [6]. Hence, the *Pf*pmt is a crucial target for structure based drug designing.

Few compounds such as sinefugin (AdoMet analog), miltefosine (Choline analog) amodiaquine (4-aminoquinoline) had been identified to inhibit the PMTs of *Plasmodium* and nematode [7–10]. Miltefosine with antitumor and antileishmanial activities also found to arrest the function of *Pf*pmt at higher concentration in radioactivity-based assay [11–13]. It was also observed that aminoquinolines and amino alcohols such as quinacrine, quinidine and quinine had no *Pf*pmt inhibitory activity at concentrations up to 200 μM [14]. Among the antimalarials chloroquine and primaquine were also found to inhibit *Pf*pmt in millimolar [7]. Eleven compounds from National Cancer Institute Open Chemical Repository were screened as *Pf*pmt inhibitor based on *Pf*pmt-specific enzyme-coupled spectrophotometric assay. These 11 compounds inhibited both *Pf*pmt and gametocytes. Compounds NSC-323241 (3-azabicyclo[3.2.2.]nonane-3-carboselenoic acid, [1-(2-pyridinyl)ethylidene]hydrazide) and NSC-158011 (*N*-naphthalen-1-yl-2-phenylsulfanylethanethioamid) found effective against early-stage gametocyte development where NSC-158011 found to inhibit the protein competitively [13] (Table 2). In this paper work we used the binding site of NSC-158011 (phosphoethanolamine/phosphocholine binding site) for screening the compound library to find the compounds with novel scaffold with better antimalarial activity.

**Table 2:** Reported inhibitors of *Pf*pmt

S. no.	Compound	(µM) *Pf*PMT activity IC50	(µM) asexual proliferation IC50
1	NSC 39225	2.4 ± 1.5	5.6
2	NSC 22225	3.1 ± 2.0	0.8 ± 1.1
3	NSC 109268	3.3 ± 1.3	2.7
4	NSC 310551	3.5 ± 1.6	3.8 ± 1.8
5	NSC 348401	3.9 ± 0.3	5.2 ± 0.2
6	NSC 641296	3.8 ± 0.7	3.6 ± 1.2
7	NSC 125034	4.3 ± 2.7	4.7
8	NSC 668394	7.7 ± 4.8	1.3 ± 0.4
9	NSC 150080	8.5 ± 4.9	4.8
10	NSC 158011	9.2 ± 3.5	5.2
11	NSC 323241	9.5 ± 3.4	1.1 ± 0.2

We carried out study for finding specific inhibitors of *Pf*pmt based on docking studies and ADMET analysis. The Natural compounds of ZINC database were shortlisted based on the Lipinski rule of five, ADMET and docking score. The final seven compounds, which were not only interacting with crucial amino acids but also occupying the catalytic dyad important for triple methylation were procured for invitro analysis. Two compounds ZINC02103914 and ZINC12882412 showed good parasite inhibition at 3.0 µM and 2.1 µM IC_50_ respectively. The Molecular dynamics (MD) simulation of best inhibitor ZINC12882412 did using Desmond *v4.2* also confirmed affinity and specificity of the inhibitor towards the *Pf*pmt. These compounds were also found nontoxic to the HEK-293 cells. Hence these compounds may further be optimized as antimalarial and may be used as template for further structure and ligand based drug designing.

## Results and discussion

### ADMET analysis of compound library

The ZINC natural compounds library before docking was subjected for computational (absorption, distribution, metabolism, excretion and toxicity) ADMET analysis. Compounds procured were fulfilling the rule of five for oral absorption. Solubility of compounds was analyzed based on solubility where levels 3 and 4 stated the good and optimum solubility. The compounds given in Table 3 were having the good solubility profile.

**Table 3:** In silico ADME and physicochemical parameters analysis of selected compounds are shown

S. no.	Structure and zinc id	RV	S	S	A	Al	P
1		0	− 2.6	3	1.5	0	114.7
2		0	− 3.7	3	2.7	0	99.8
3		0	− 3.6	3	2.8	0	107.1
4		0	− 3.3	3	2.0	0	104.8
5		0	− 3.0	3	2.5	0	94.7

(ALogp98) value should range between the − 2.0 and 5.0 and PSA should not be more than 140 A for good intestinal absorption (as per Lipinski rule)

*RV* rule of violation, *S* solubility, *SL* solubility level, *A* AlogP98, *AL* absorption level, *P* PSA

The absorption profile was also estimated based on the stability between lipophilicity (Alogp98) and polar surface area (PSA) of the compounds. The absorption level 0 stated the good absorption as given in Table 3.

Hence, the compounds may be having the significant probability of good pharmacokinetic profile for orally absorption. Toxicity analysis, confirmed the non-hepatotoxicity, noncarcinogenicity, and nonmutagenicity of the selected compounds. There no affinity was also found against Cyp2d6 (cytochrome enzyme) found revealed that there is no possibility of drug–drug interaction (Table 4).

**Table 4:** Shows predictions for the affinity of compounds with CyP2D6, plasma protein binding as well as toxic physicochemical properties of procured compounds

S. no.	Zinc id	Cyp2d6 prediction	Ames prediction	Carcinogen prediction	Toxicity	Hepto toxicity	Plasma protein binding
1	ZINC08792082	False	Non mutagen	Non carcinogen	Nontoxic	False	False
2	ZINC02120366	False	Non mutagen	Non carcinogen	Nontoxic	False	False
3	ZINC08792474	False	Non mutagen	Non carcinogen	Nontoxic	False	False
4	ZINC12882412	False	Non mutagen	Non carcinogen	Nontoxic	False	False
5	ZINC02103914	False	Non mutagen	Non carcinogen	Nontoxic	False	False

### Docking analysis of compounds and schizonticidal activity

Compounds with good ADMET properties were selected for docking analysis. In order to find better and more thermodynamically stable compounds, the compounds with docking score and binding energy more than the pCholine docking score (51.8 kcal/mol) and binding energy (− 89.5 kcal/mol) were selected. The compounds were docked with *Pf*pmt and the top twenty compounds with dock score and binding energy more than 60 kcal/mol and − 90.2 kcal/mol respectively were selected for interaction analysis. Selection of compounds was done based on better interaction with *Pf*pmt. Compounds showed interaction with crucial amino acids like residues Tyr19 and His132 which are important for formation of catalytic dyad and other tyrosine residues important for transmethylation of phosphoethanolamine.

**Fig. 2:**
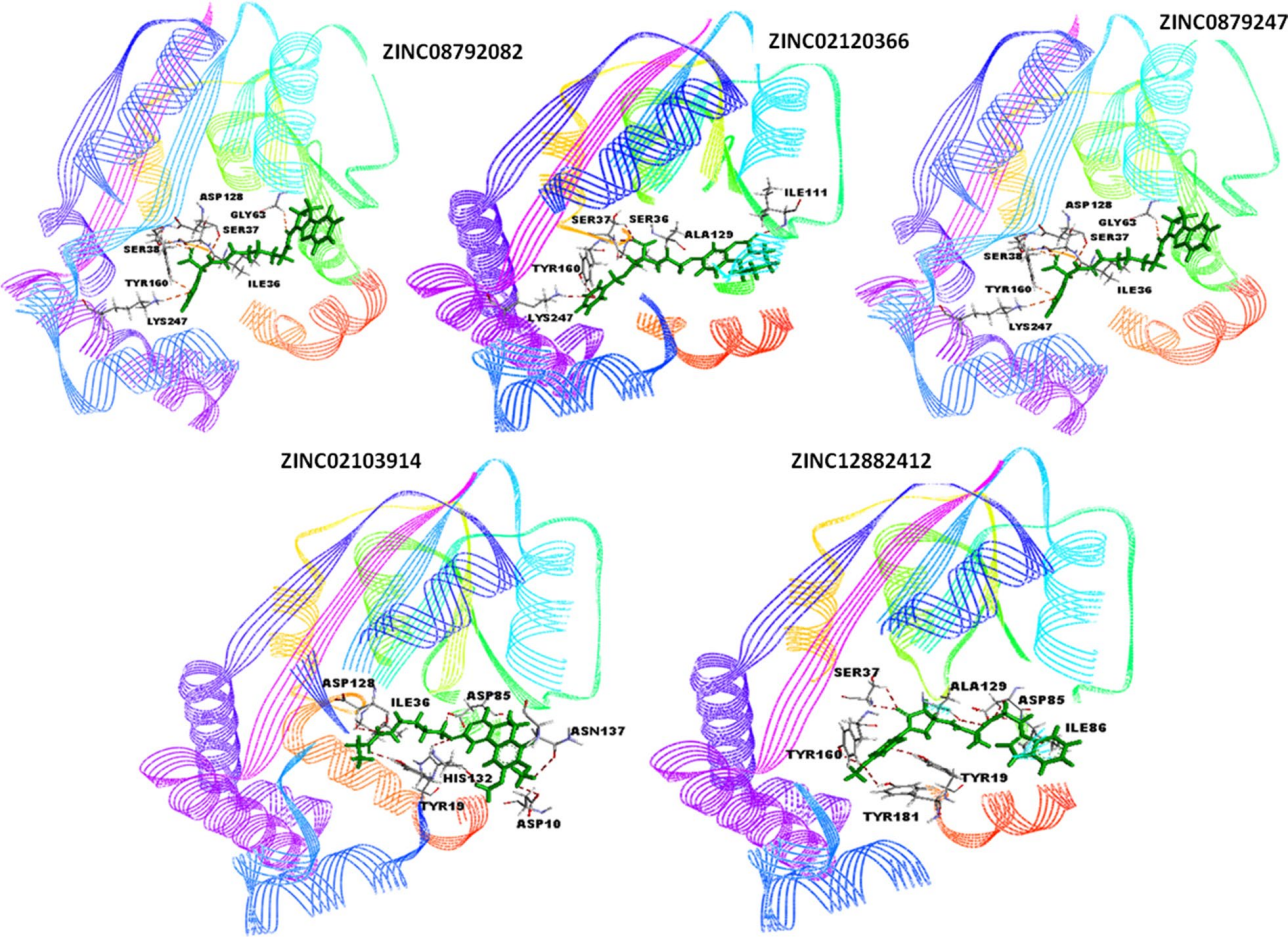
Interaction of compounds (green color) within the active pocket and interacting amino acids

All selected compounds formed hydrogen bonds with crucial amino acids of pCholine binding pocket importantly, with Tyrosine residues. The significantly compounds inhibitors ZINC08792474, ZINC12882412 and ZINC02103914 occupied dyad between Tyr19, His132 crucial for triple methylation of pEth. Top Glide XP scoring compound ZINC08792082 formed H-bond interaction with Tyr160 important residue of pCholine site as shown in Fig. 2. All the seven compounds selected based on interaction with the important amino acids which may be preventing the formation of catalytic dyad and binding (Table 5) of pEth for transmethylation were procured and tested against trophozoites (Schizont) stage of *Plasmodium falciparum*. Compound showed schizonticidal activity at less than five micromolar concentrations were selected as potent antimalarial compounds. Two compounds ZINC12882412 and ZINC02103914 could inhibit schizonts formation at 2.1 µM and 3.0 µM concentration, while the negative control (chloroquine) inhibited the formation of schizonts at 0.069 µM which suggested that the identified two are also active and may as potential antimalarial drug.

**Table 5:** Docking score, binding energy and interacting amino acids of selected compound

S. no.	Zinc id	Dock score	Binding energy	Interacting amino acids form (pCholine binding site)
1	ZINC08792082	82.3	− 98.2	Ser 38, Asp 128, Tyr 160, Lys 247, Ile 36, Ser 37, Gly 63
2	ZINC02120366	79.67	− 102.3	Ala 129, Tyr 160, Lys 247, Ile 36, Ser 37, Ile 111
3	ZINC08792474	89.67	− 98.4	Tyr 19, Asp 128, Gly 63, Asp 85
4	ZINC12882412	108.80	− 103.5	Tyr 19, Ile 86, Ala 129, Tyr 160, Tyr 181, Ser 37, Asp 85
5	ZINC02103914	98.51	− 110.9	Asp 10, Tyr 19, Asp 128, Asn137, Ile 36, Asp 85, His 132

**Fig. 3:**
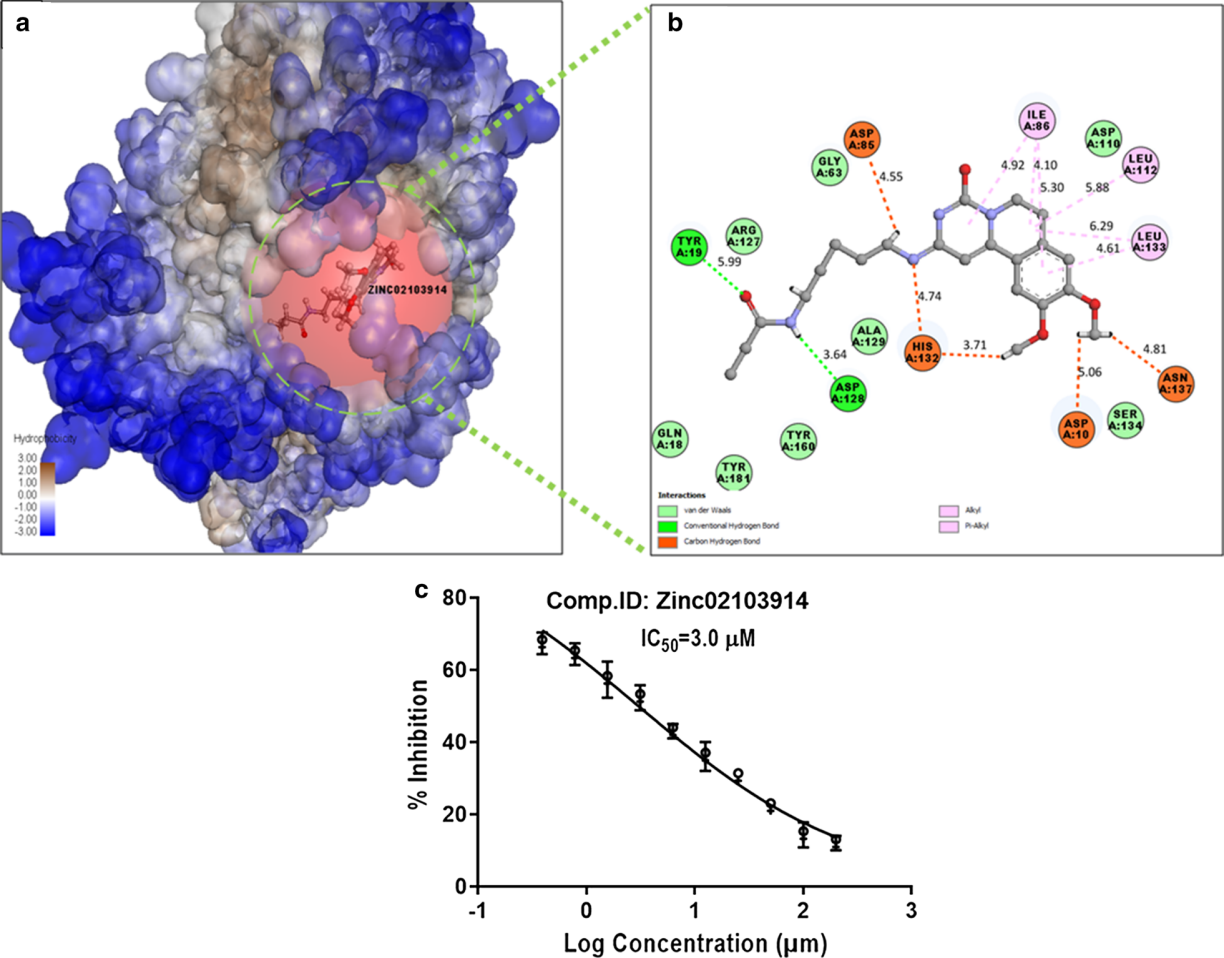
Interaction of compounds ZINC02103914. **a** Orientation in the hydrophobic surface view, **b** 2D interaction within the binding pocket, **c** schizonticidal activity (IC_50_) of the ZINC02103914 compound

Compound ZINC02103914 which scored good docking score (98.51 kcal/mol) occupied pCholine binding pocket by forming hydrogen bonds with Tyr19 and Asp128 where Tyr19 acted as H-donor provided side chain hydrogen to the double bonded oxygen of compound to form H-bond. Asp128 as H-acceptor provided side chain double bonded oxygen to the NH group of compound to form H-bond. Compound also formed Van der Waals interaction with crucial tyrosine residues like Tyr160, Tyr181 and also formed carbon hydrogen bonds with His132 which are very crucial for transmethylation. The heterocyclic ring pyrido-pyrimidine-1-one fused with benzene ring was found actively involved in the interaction and the aliphatic chain was also found to interact with crucial amino acids implied the importance of pyrido-pyrimidine-1-one in keeping compound intact within the active pocket and thermodynamic stability (Fig. 3). Compound ZINC02103914 also inhibited the *Plasmodium falciparum* schizonts formation at very low micromolar concentration IC_50_ 3.0 µM which implied the good antimalarial potency of the compound.

**Fig. 4:**
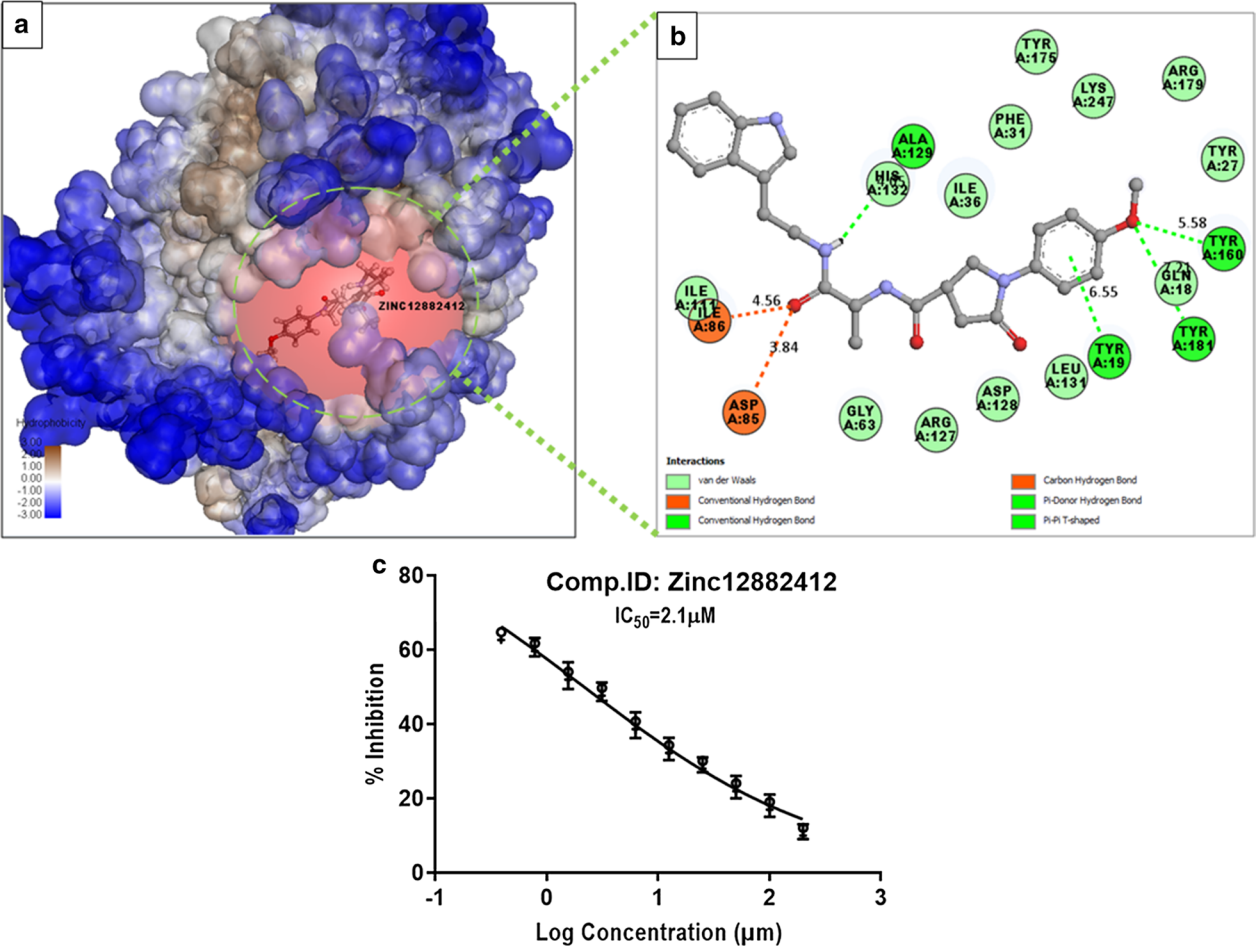
Interaction of compounds ZINC12882412. **a** Orientation in the hydrophobic surface view, **b** 2D interaction within the binding pocket, **c** schizonticidal activity (IC_50_) of the ZINC12882412 compound

The compound ZINC12882412 also showed good docking score (108.80 kcal/mol) than pCholine. Compound ZINC12882412 provided four hydrogen bonds with Tyr19, Tyr181, Tyr160 and Ala129 where binding with these three tyrosine residues showed the better affinity for *Pf*pmt. Both the amino acids (Tyr160 and Tyr181) acted as H-bond donor and provided side chain hydrogens to the compound’s hydroxyl oxygen. Compound also participated in the Van der Waals interaction with amino acids like His132 and Lys247 crucial for catalytic dyad formation and protein function. The binding with tyrosine residues increased the probability of inhibition of transmethylation. Compound ZINC12882412 possessed pyrrole and pyrrolidine heterocyclic rings fused with benzene rings at both ends of the it which may be crucial for the stability and activity of the compound which could inhibit the formation of schizonts at IC_50_ 2.1 µM which showed the potency of the compound (Fig. 4). Very low micromolar concentration of these compounds implied the good potency and specificity of compounds for *Pf*pmt as inhibitors.

Hence, these two compounds may act as potent inhibitors for *Pf*pmt. Since *Pf*pmt has significant identity with other pmt *Plasmodium* orthologs so, the inhibitors may also be effective antimalarial worldwide.

### Cytotoxicity testing

**Fig. 5:**
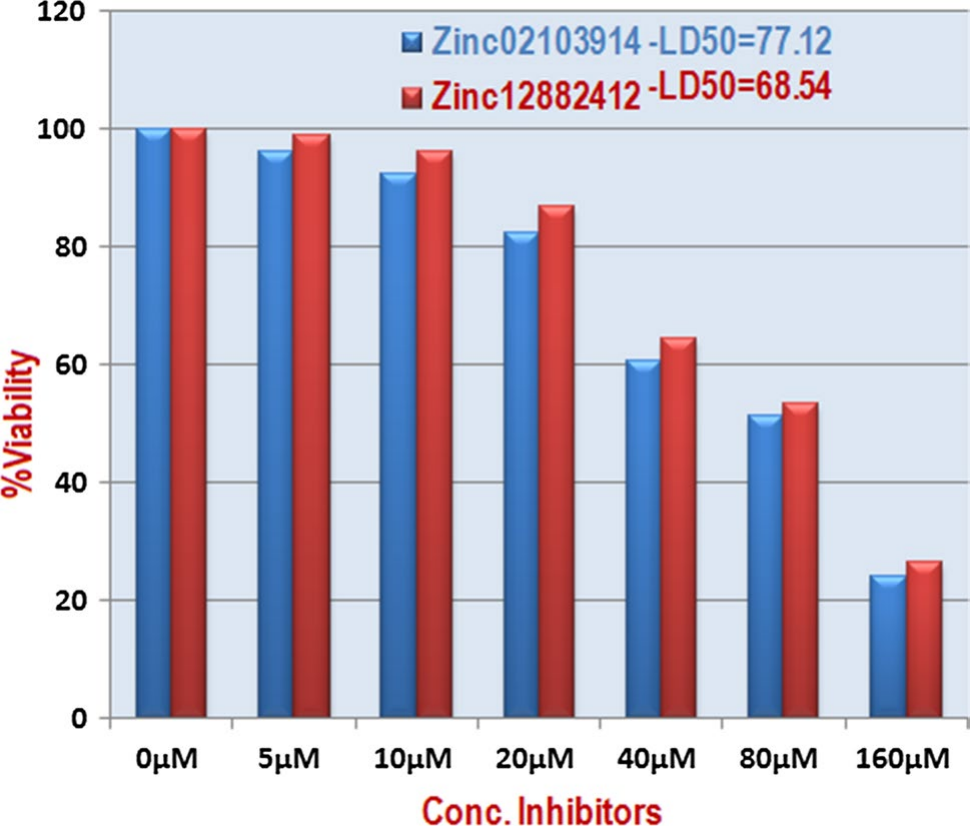
Showed the toxicity analysis of the different concentrations of compounds against the HEK-293 cells

Five thousand HEK-293 cells were embedded with both the selected compounds diluted in concentrations (5 µM, 10 µM, 20 µM, 40 µM, 80 µM, 1600 µM) for testing Fig. 5. Both the compounds ZINC02103914 and ZINC12882412 were found safer with LD_50_ concentration 77.12 µM and 68.54 µM respectively. Even the higher concentration than the IC_50_ was found nontoxic for the HEK-293 cells. Both the compounds ZINC02103914 and ZINC12882412 attained the better safety index 25.83 µM and 32.63 µM respectively Table 6.

**Table 6:** Cytotoxicity of compounds with Schizonticidal compounds against HEK-293 cells

S. no.	Comp. id	IC_50_ schizonticidal (µM)	LD_50_ cyto-toxicity (µM)	Safety index (LD_50_/IC_50_)
1	ZINC02103914	3.0	77.12	25.83
2	ZINC12882412	2.1	68.54	32.63

The compounds were also found nontoxic noncarcinogenic and nonmutagenic in computational toxicity predictions which correlated with invitro. Hence, the better safety index of the compounds implied about therapeutic safety that these may be safer within the body.

### Dynamic simulation of protein–ligand complex

In order to confirm binding modes and interaction of best *Pf*pmt inhibitor (ZINC12882412), the molecular dynamic simulation (MDS) was run using Desmond. The behavior of the protein and inhibitor was studied in terms of root mean square deviation (RMSD) and root mean square fluctuation between protein and ligand and protein–ligand interactions throughout the simulations.

#### Root mean square deviation

**Fig. 6:**
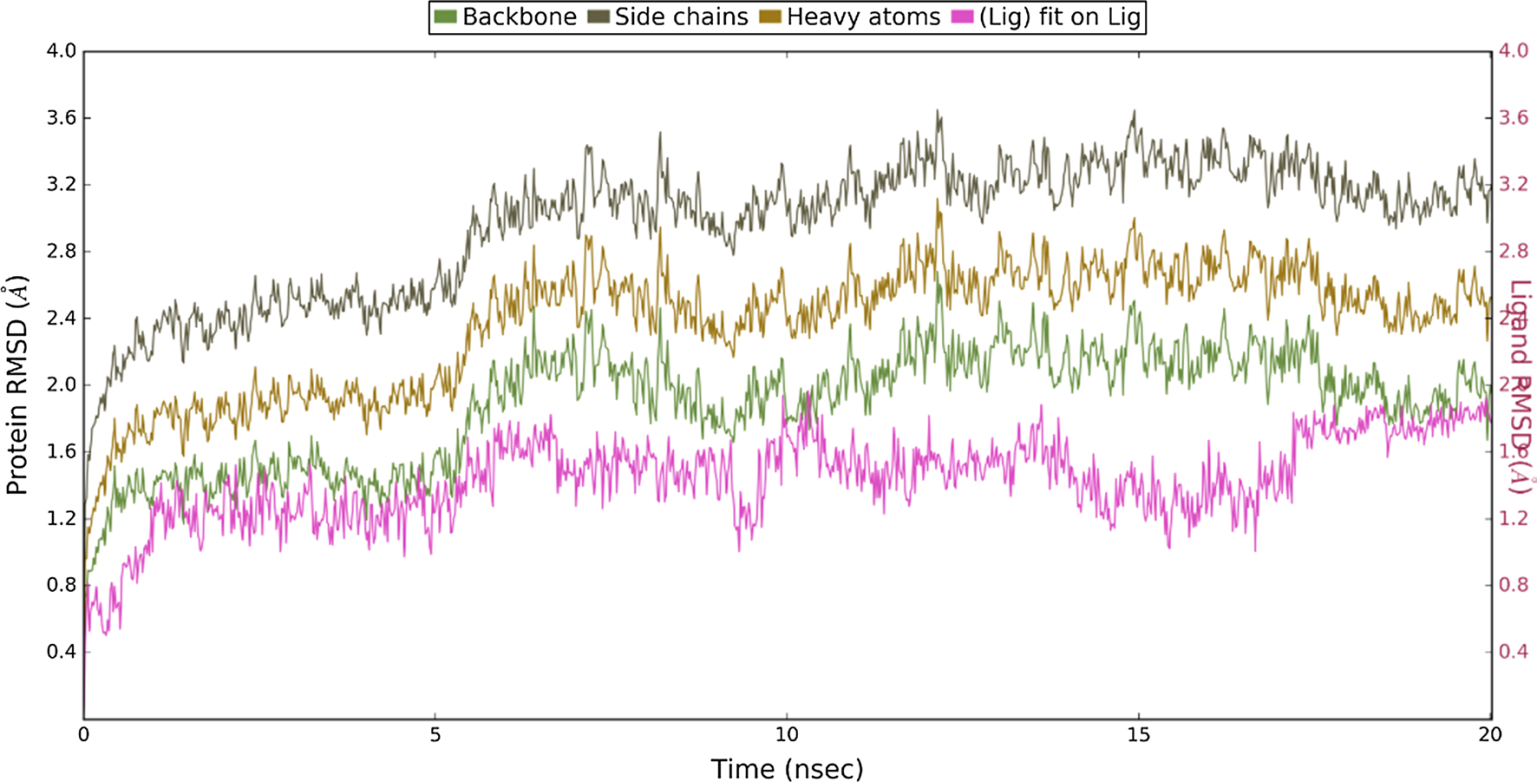
Protein–ligand RMSD. It showed the RMSD between the backbone, side chain and heavy atoms aligned on the ligand

To explore the dynamic stability of systems, MD properties were observed up to 20 ns for protein–ligand complex and RMSD values over time from the starting structure was analyzed as depicted in Fig. 4. The MD analysis of protein–ligand complex showed that the RMSD trajectories within the allowed range (1–3 Å) for small, globular proteins. From 0 to 5 ns system showed slight fluctuation to equilibrate with the external environment and after 5 ns, the system remained equilibrated at the end of simulation that give insight into its stable structural conformation. The backbone of protein showed not as much of deviation as side chain, this might be due to interaction of ligand with the flexible side chain residues (Fig. 6).

The less RMSD of Lig fit Prot’ as compared to the RMSD of protein, demonstrated the stability of inhibitor interactions with protein as the movement of inhibitors remained within the binding pocket throughout the simulation. Throughout the dynamic simulation the stability of protein was remained constant but there little RMSD fluctuations were observed in the inhibitors due to conformational adjustment and interaction with new amino acids within the active site.

#### Root mean structure fluctuation

**Fig. 7:**
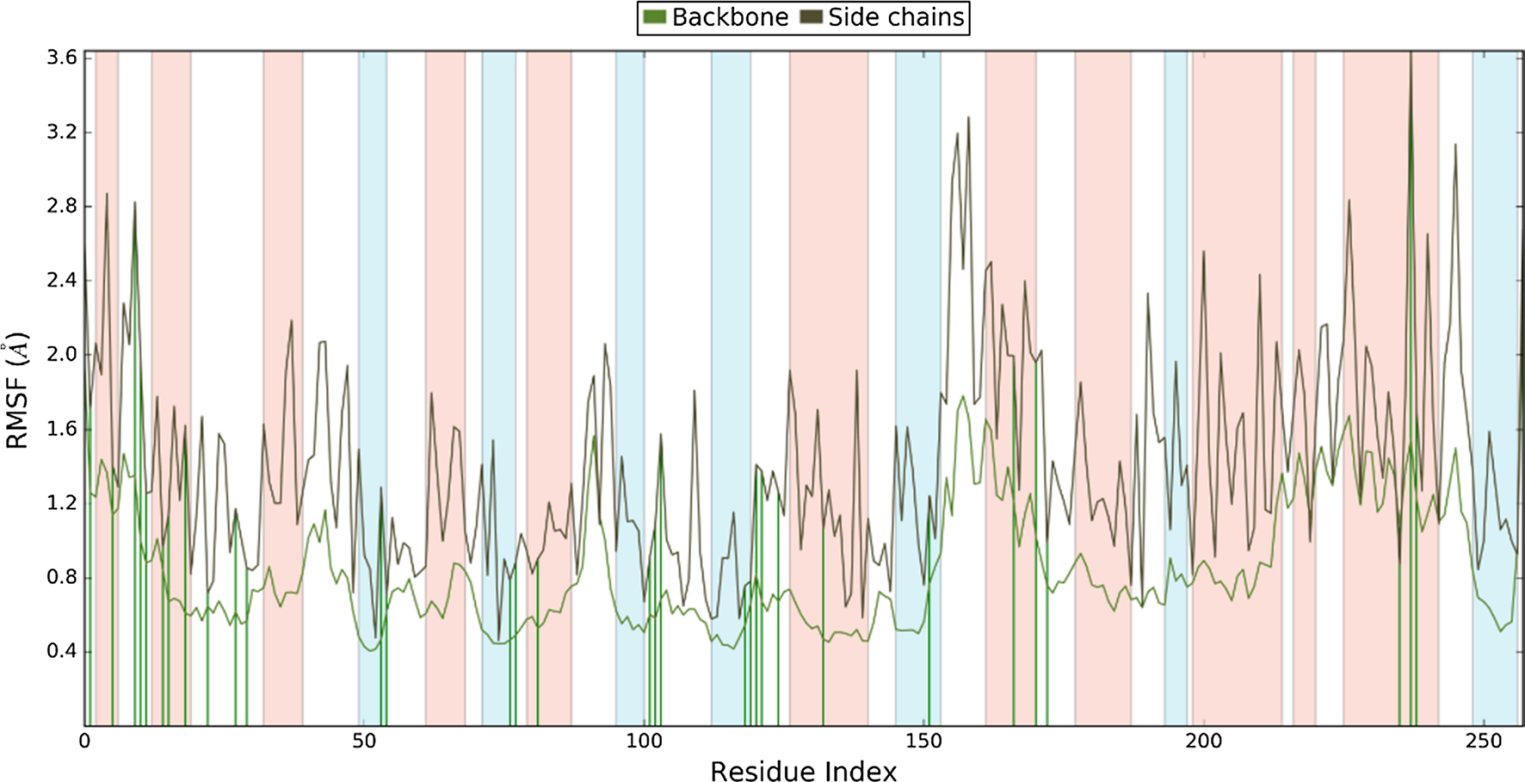
Protein RMSF: showed the fluctuations among the backbone and the sidechains during the simulation. The green lines are amino acids participated in interacting with ligand throughout the simulation

Root mean structure fluctuation (RMSF) of protein–ligand complex was monitored throughout the simulation period that indicated that the secondary structure elements like alpha helices and beta strands are usually more rigid than the unstructured part of the protein. Minor fluctuations were seen in the active site pocket (residue 80–100) and (residue 150–250) that indicate that the active site residues may be fluctuating in order to form interaction with the ligand (Fig. 7).

#### Protein ligand contacts

**Fig. 8:**
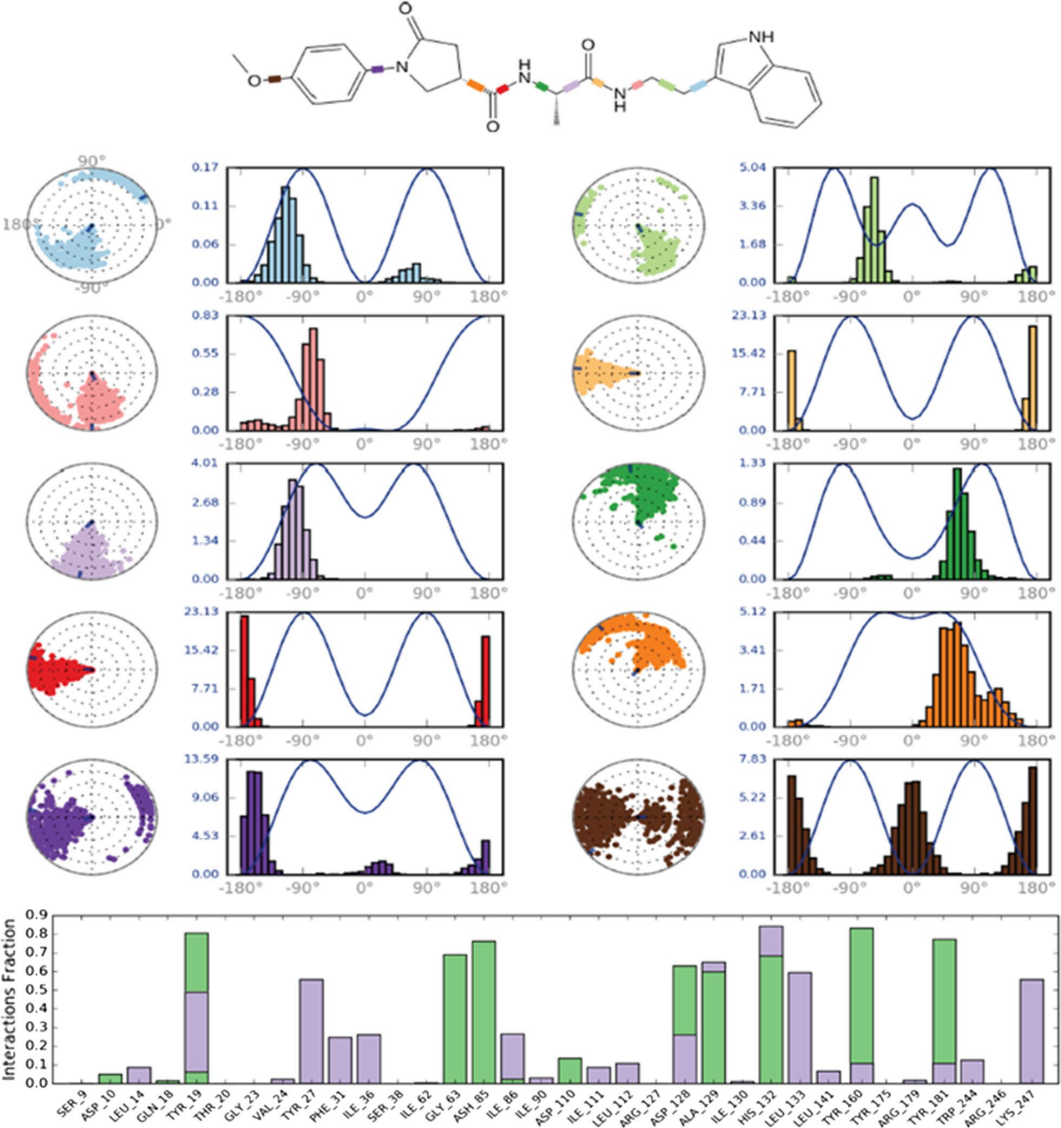
Ligand torsion plot: conformational changes within the rotatable bonds throughout the simulation. The protein–ligand interaction (green: hydrogen bond, purple: hydrophobic) is represented by respective colors as shown in the box

Interaction of protein with the ligand throughout the simulation has been explained in protein ligand contacts. The compound ZINC12882412 had ten rotatable bonds which undergone continuous conformational adjustments throughout the simulation. Protein–ligand interactions (or ‘contacts’) categorized into four types: hydrogen bonds, hydrophobic, ionic and water bridges. We found, that the inhibitors had stable H-bond interaction with the active site residues that had initial interaction (Tyr 19, Ile 86, Ala 129, Tyr 160, Tyr 181, Ser 37, and Asp 85) as H-bond interactions are important because of their strong influence on drug specificity, metabolism and adsorption. We also observed interaction of other residues within the binding pocket that may be due to flexibility of ligand in presence of water shown in Fig. 8.

### Experimental

#### Ethics approval and consent to participate

This study was conducted under the approval of the Human Ethical Committee of National Institute of Malaria Research (NIMR), New Delhi (Approval No. ECR/NIMR/EC/2015/206) with proper written documentation of informed consent. The consent was also explained verbal to the patient. The minors were excluded from study.

#### Compound library and ADMET analysis

Compound library was made from Natural compound of ZINC database. The ADMET that is computational analysis of pharmacokinetic properties of a compound was calculated from ADMET and toxicity prediction (TOPKAT) modules of discovery Studio 3.5 (DS3.5) [15]. The compounds, devoid of ADME descriptors and Lipinski criteria were removed at very start to save experimental cost and time [16, 17]. Carcinogenicity, mutagenicity and affinity for Cytochrome P450 2D6 (Cyp2d6) were also analyzed. DS3.5 is robust software for inslico ADME and toxicity analysis [18].

#### Docking analysis of compounds

The crystal structure of *Pf*pmt 3D7 (PDB Id: 3UJ9) was retrieved from PDB (http://www.rcsb.org/pdb/home/home.do) for docking studies [19]. The protein was prepared by applying CHARMm forcefield using DS*3.5* The binding site was created around the co-crystallized phosphocholine (pCholine) using Discovery Studio *3.5*. The binding site covered the important interacting amino acids with pCholine to screen the compounds. Docking of the filtered compound library was carried out using LibDock docking module of DS*3.5* which works based on formation of polar and apolar HotSpot [20, 21]. The compounds were scored based on the orientation of compounds within the binding site and docking score. The binding energy was also calculated. The pCholine and pEth were used as docking control in the study so; these were also docked with *Pf*pmt.

#### Compound preparation and invitro schizonticidal activity

Parasite strain *Plasmodium falciparum 3D7* available at parasite bank, National Institute of Malaria Research, India was grown under optimum conditions. RPMI1640 media was used as growth medium for parasite. Other required growth supplements like glucose and antibiotic were added to the RPMI1640 media at the time of media preparation. Incomplete media was prepared with 5% sodium bicarbonate and complete media was prepared by adding human serum (10% heat inactivated) to the incomplete media. The human serum was collected from Indian Red Cross Society-1, Red Cross Road New Delhi-11000, INDIA. When rings formed, parasite culture was treated with 5% sorbitol and rings were separated. Parasite 1% rings were used for schizonticidal activity of compounds in 96 well-microlitre plate [22].

For each compound the stock solution of 200 µM was prepared in 0.1% DMSO and ten serial dilutions of each were prepared. Experiments were performed in triplicates with controls and reference drug (chloroquine) by placing it in CO_2_ incubator at 37 °C. After 32–36 h when schizonts formed, the blood Giemsa smear glass slides were prepared for each well of control and sample for microscopic evaluation. The schizonts were counted from 200 asexual parasites microscopically and percent parasitaemia calculated.

#### Cytotoxicity testing

MTT (3-[4,5-dimethylthiazol-2-yl]-2,5-diphenyltetrazolium bromide) based cytotoxicity testing of two compounds was done against Human embryonic kidney cells 293 (HEK293). The HEK 293 cells of human were procured from ATCC with identifier no. 293 [HEK-293] (ATCC^®^ CRL-1573™) and catalog no CRL-1573. The cells were grown in Dulbecco’s Modified Eagle’s Medium. Growth medium was supplemented with glucose, penicillin and fetal bovine serum (5%). Washed and trypsinized five thousand HEK-293 grown cells were seeded with containing growth media into 96-well plate and cell viability was checked after 24 h using trypan blue dye exclusion test. Cells showed viability 98% were incubated with different concentration of primary hits to the 96 well plate for 24 h followed by 4 h incubation with MTT 5 mg/ml. The absorbance was recorded at 580 nm using Synergy/HTX MultiScan reader (BioTek) [23]. Experiments were carried out in triplicate and the Lethal dose (LD_50_) of each primary hits was calculated and the safety index (SI) was calculated.

### Dynamics simulation

Molecular dynamics (MD) simulations were done to analyze more intrinsically the binding mode stability and interaction pattern of best identified inhibitor through docking studies. MD simulation was run using the Desmond *v 4.2* [24]. The protein was prepared for by adding hydrogen, assigning bond orders and by generating protonation states at physiological pH (7.4) for MD run, thereafter, the missing side chain and missing loop were filled and terminal residues were capped with neutral groups such as N-terminal acetyl and C-terminal amide and protein was optimized. Then, the protein–ligand complex system was solvated with TIP3P water model inside the orthorhombic box ensuring 10 Å solvent buffers and system was neutralized by adding Na^+^ and Cl^−^ ions and simulations were assigned using the OPLS-AA/2005 force field. Then, the solvated system was relaxed using relaxation protocol as implemented in Desmond. Finally, a 20 ns production simulation was performed using the NPT ensemble using Nose–Hoover chain thermostat and Martyna–Tobias–Klein with isotropic coupling at constant temperature 300 K and pressure of 1ATM. The long-range electrostatic interactions were handled using the particle-mesh Ewald (PME) method. A distance cutoff of 9.0 Å was used for short-range electrostatics and Lennard–Jones interactions. Energy and trajectory were saved at every 10 ps and 20 ps time interval respectively. The trajectory for RMSD and interaction of ligand with protein was visualized in Maestro [25].

## Conclusion

Phosphatidylcholine an important component of membrane which is synthesized abundantly from serine decarboxylation pathway during intraerythrocytic cycle as well as gametocyte development cycles of *Plasmodium.* The knockout of *Pf*pmt revealed importance of *Pf*pmt in the phosphatidylcholine biosynthesis from serine for survival and malaria transmission and moreover absence of *phosphoethanolamine methyltransferse* in human makes this enzyme an excellent drug target for development of new antimalarial. The compound library built from natural compound of zinc database was screened based on the docking, Lipinski rule of five and ADMET analysis. Compounds were selected based on the good docking scoring and ADMET physicochemical properties. Five compounds (ZINC08792082, ZINC02120366, ZINC08792474, ZINC12882412 and ZINC02103914) found better based on *insilico* analysis and procured for invitro testing. These compounds formed the hydrogen interaction with crucial tyrosine residues and also crucial amino acids of pCholine binding site. The schizonticidal activity was done against the 1% rings of *Plasmodium falciparum* where best compounds found ZINC12882412 (IC_50_ 2.1 µM) and ZINC02103914 (IC_50_ 3.0 µM) were occupied with catalytic dyad between Tyr19 and His132. These two compounds inhibited the development of schizonts from rings at very low µM concentration (3.0 µM and 2.1 µM) implied probability of being occupied with the crucial amino acids of target protein to prevent its biological function and the non-toxicity towards the kidney cells confirmed the safety of these two compounds too. The simulation study of 3*D* binding mode of the best inhibitor ZINC12882412 elucidated that the binding with the important active site residues were also conserved throughout the MD simulation for 20 ns that signified the stability of interaction near the active site. Hence, these compounds may act as potent inhibitors of *Pf*pmt and may lead as template for structure based drug designing to overcome the problem of multi drug resistance.

## Abbreviations

*Pf*pmt: *Plasmodium falciparum* phosphoethanolamine methyltransferase
HEK-293: human embryonic kidney 293 cells
ADMET: absorption distribution metabolism excretion and toxicity
ACT: artemisinin combination therapy
DAG: diacylglycerol
CDP: cytidine diphosphate
PC: phosphatidylcholine
PSA: polar surface area
Cyp2d6: cytochrome enzyme
MD: molecular dynamic
RMSD: root mean square deviation
RMSF: root mean structure fluctuation
DMSO: dimethyl sulfoxide
OPLS-AA: optimized potentials for liquid simulations
NPT: normal pressure and temperature

## References

[1] World Health Organization (2015). World malaria report 2014.

[2] World Health Organization (2015). Guidelines for the treatment of malaria.

[3] Dash A, Valecha N, Anvikar AR, Kumar A (2008). Malaria in India: challenges and opportunities. J Biosci.

[4] Reynolds JM, Takebe S, Choi JY, El Bissati K, Witola WH, Bobenchik AM (2008). Biochemical and genetic analysis of the phosphoethanolamine methyltransferase of the human malaria parasite *Plasmodium* falciparum. J Biol Chem.

[5] Gabriella P, Jae YC, Jennifer MR, Dennis RV, Choukri BM (2005). *In vivo* evidence for the specificity of *Plasmodium falciparum* phosphoethanolamine methyltransferase and its coupling to the Kennedy pathway. J Biol Chem.

[6] Egan WJ, Merz KM, Baldwin JJ (2000). Prediction of drug absorption using multivariate statistics. J Med Chem.

[7] Nuccio M, Ziemak MJ, Henry SA, Weretilnyk EA, Hanson AD (2000). cDNA cloning of phosphoethanolamine *N*-methyltransferase from spinach by complementation in Schizosaccharomyces pombe and characterization of the recombinant enzyme. J Biol Chem.

[8] Bobenchik AM, Jae-Yeon C, Arunima M, Iulian NR, Bing H, Dennis RV (2010). Identification of inhibitors of *Plasmodium falciparum* phosphoethanolamine methyltransferase using an enzyme-coupled transmethylation assay. BMC Biochem.

[9] Lee SG, Haakenson W, McCarter JP, Williams DJ, Hresko MC, Jez JM (2011). Thermodynamic evaluation of ligand binding in the plant-like phosphoethanolamine methyltransferases of the parasitic nematode *Haemonchus contortus*. J Biol Chem.

[10] Lux H, Heise N, Klenner T, Hart D, Opperdoes FR (2000). Ether–lipid (alkyl-phospholipid) metabolism and the mechanism of action of ether–lipid analogues in Leishmania. Mol Biochem Parasitol.

[11] Jha TK, Sundar S, Thakur CP, Bachmann P, Karbwang J, Fischer C (1999). Miltefosine, an oral agent, for the treatment of Indian visceral leishmaniasis. N Engl J Med.

[12] Croft SL, Seifert K, Duchene M (2003). Antiprotozoal activities of phospholipid analogues. Mol Biochem Parasitol.

[13] Bobenchik AM, Witola WH, Augagneur Y, Nic Lochlainn L, Garg A, Pachikara N (2013). *Plasmodium falciparum phosphoethanolamine methyltransferase* is essential for malaria transmission. Proc Natl Acad Sci USA.

[14] Witola WH, El Bissati K, Pessi G, Xie C, Roepe PD, Mamoun CB (2008). Disruption of the *Plasmodium falciparum Pf*pmt gene results in a complete loss of phosphatidylcholine biosynthesis via the serine-decarboxylase-phosphoethanolamine-methyltransferase pathway and severe growth and survival defects. J Bio Chem.

[15] Cheng A, Merz KM (2003). Prediction of aqueous solubility of a diverse set of compounds using quantitative structure–property relationships. J Med Chem.

[16] Gombar VK, Enslein K (1996). Assessment of n-octanol/water partition coefficient: when is the assessment reliable?. J Chem Inf Comput Sci.

[17] Yamini L, Vijjulatha M (1996). Inhibitors of human dihydrofolate reductase: a computational design and docking studies using glide. J Chem Inf Comput Sci.

[18] Lee SG, Kim Y, Alpert TD, Nagata A, Jez JM (2012). Structure and reaction mechanism of phosphoethanolamine methyltransferase from the malaria parasite *Plasmodium falciparum* an antiparasitic drug target. J Biol Chem.

[19] Diller DJ, Merz KM (2001). High throughput docking for library design and library prioritization. Proteins.

[20] Singh J, Kumar M, Mansuri R, Sahoo GC, Deep A (2016). Inhibitor designing, virtual screening, and docking studies for methyltransferase: a potential target against *dengue virus*. J Pharm Bioallied Sci.

[21] Wadi I, Pillai CR, Anvikar AR, Sinha A, Nath M, Valecha N (2018). Methylene blue induced morphological deformations in *Plasmodium* falciparum gametocytes: implications for transmission-blocking. Malar J.

[22] Selvaraj V, Bodapati S, Murray E, Rice KM, Winston N, Shokuhfar T (2014). Cytotoxicity and genotoxicity caused by yttrium oxide nanoparticles in HEK293 cells. Int J Nanomed.

[23] Kuenemann MA, Fourches D (2018). Cheminformatics analysis of dynamic WNK–inhibitor interactions. Mol Inform.

[24] Ash J, Fourches D (2017). Characterizing the chemical space of ERK2 kinase inhibitors using descriptors computed from molecular dynamics trajectories. J Chem Inf Model.

[25] Mansuri R, Kumar A, Rana S, Panthi B, Ansari MY, Das S (2017). In vitro evaluation of antileishmanial activity of computationally screened compounds against ascorbate peroxidase: combating amphotericin B drug resistance. Antimicrob Agents Chemother.

